# Ethyl (*E*)-2-methoxy­imino-2-(4-nitro­benzo­yl)acetate

**DOI:** 10.1107/S1600536810001583

**Published:** 2010-01-20

**Authors:** Ignez Caracelli, Paulo J. S. Moran, Luciana Hinoue, Julio Zukerman-Schpector, Edward R. T. Tiekink

**Affiliations:** a BioMat-Physics Department, UNESP – Univ Estadual Paulista, 17033-360 Bauru, SP, Brazil; b Instituto de Química, Universidade Estadual de Campinas, CP 6154, 13083-970 Campinas, SP, Brazil; c Department of Chemistry, Universidade Federal de São Carlos, 13565-905 São Carlos, SP, Brazil; d Department of Chemistry, University of Malaya, 50603 Kuala Lumpur, Malaysia

## Abstract

The title mol­ecule, C_12_H_12_N_2_O_6_, features an *E* conformation about the oxime group. The methoxy­imino and ester residues are effectively coplanar with each other (r.m.s. deviation for the nine non-H atoms = 0.127 Å) and almost orthogonal [with dihedral angles of 99.44 (13) and −77.85 (13)°, respectively] to the carbonyl and nitro­phenyl groups which lie to either side of this central plane. The crystal structure is consolidated by C—H⋯O contacts.

## Related literature

For background to the synthesis of chiral hydroxy­amino­acids and hydroxy­amino­alcohols, see: Corrêa & Moran (1999 ▶); Kreutz *et al.* (1997 ▶, 2000 ▶). For related structures, see: Caracelli *et al.* (2010 ▶); Forsyth *et al.* (2006 ▶); Ramos Silva *et al.* (2004 ▶). For the synthesis of the title compound, see: Buehler (1967 ▶).
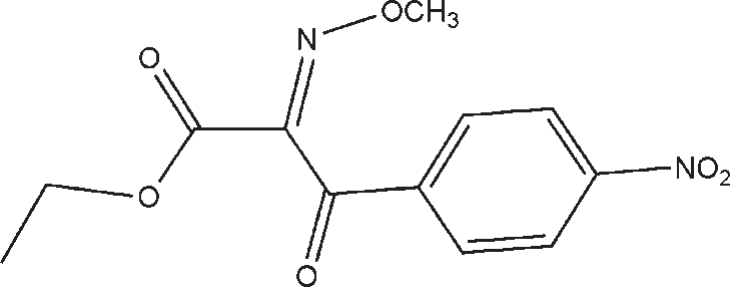


## Experimental

### 

#### Crystal data

C_12_H_12_N_2_O_6_
*M* *_r_* = 280.24
Triclinic, 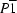

*a* = 7.5197 (1) Å
*b* = 7.5793 (1) Å
*c* = 12.3338 (2) Å
α = 83.264 (1)°
β = 73.731 (1)°
γ = 68.939 (1)°
*V* = 629.62 (2) Å^3^
*Z* = 2
Mo *K*α radiation
μ = 0.12 mm^−1^
*T* = 100 K
0.35 × 0.25 × 0.08 mm

#### Data collection

Bruker APEXII CCD diffractometer
Absorption correction: multi scan (*SADABS*; Sheldrick, 1996 ▶) *T*_min_ = 0.933, *T*_max_ = 1.000
9325 measured reflections
2614 independent reflections
2310 reflections with *I* > 2σ(*I*)
*R*_int_ = 0.018

#### Refinement

*R*[*F* ^2^ > 2σ(*F* ^2^)] = 0.031
*wR*(*F* ^2^) = 0.088
*S* = 1.05
2614 reflections
183 parameters
H-atom parameters constrained
Δρ_max_ = 0.28 e Å^−3^
Δρ_min_ = −0.24 e Å^−3^

### 

Data collection: *APEX2* (Bruker, 2007 ▶); cell refinement: *SAINT* (Bruker, 2007 ▶); data reduction: *SAINT*; program(s) used to solve structure: *SIR97* (Altomare *et al.*, 1999 ▶); program(s) used to refine structure: *SHELXL97* (Sheldrick, 2008 ▶); molecular graphics: *ORTEP-3* (Farrugia, 1997 ▶) and *DIAMOND* (Brandenburg, 2006 ▶); software used to prepare material for publication: *WinGX* (Farrugia, 1999 ▶), *PARST* (Nardelli, 1995 ▶) and *publCIF* (Westrip, 2010 ▶).

## Supplementary Material

Crystal structure: contains datablocks I, global. DOI: 10.1107/S1600536810001583/su2157sup1.cif

Structure factors: contains datablocks I. DOI: 10.1107/S1600536810001583/su2157Isup2.hkl

Additional supplementary materials:  crystallographic information; 3D view; checkCIF report

## Figures and Tables

**Table 1:** Hydrogen-bond geometry (Å, °)

*D*—H⋯*A*	*D*—H	H⋯*A*	*D*⋯*A*	*D*—H⋯*A*
C2—H2⋯O4^i^	0.93	2.56	3.3853 (14)	148
C3—H3⋯O2^ii^	0.93	2.50	3.3950 (16)	162
C5—H5⋯O5^iii^	0.93	2.35	3.1856 (14)	150
C6—H6⋯O3^iv^	0.93	2.46	3.3514 (16)	160

Symmetry codes: (i) 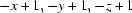; (ii) 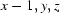; (iii) 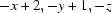; (iv) 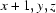.

**Fig. 1.**
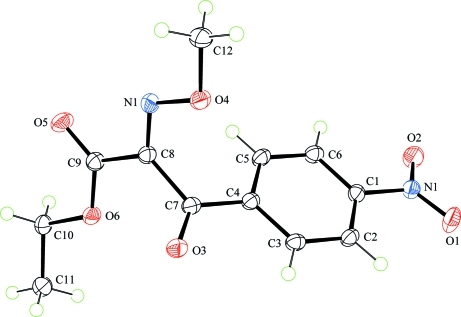
Molecular structure of the title compound, showing the atom labelling scheme and displacement ellipsoids at the 50% probability level (arbitrary spheres for the H atoms).

**Fig. 2.**
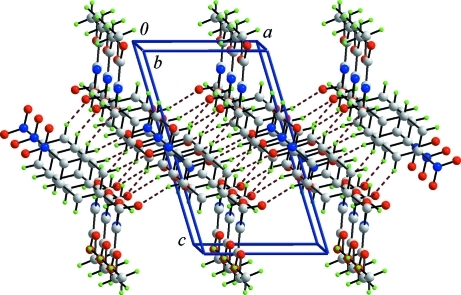
A view of the layer structure in the title compound, sustained by C–H···O contacts [the C–H···O contacts are shown as brown dashed lines; colour code: O, red; N, blue; C, grey; H, green].

**Fig. 3.**
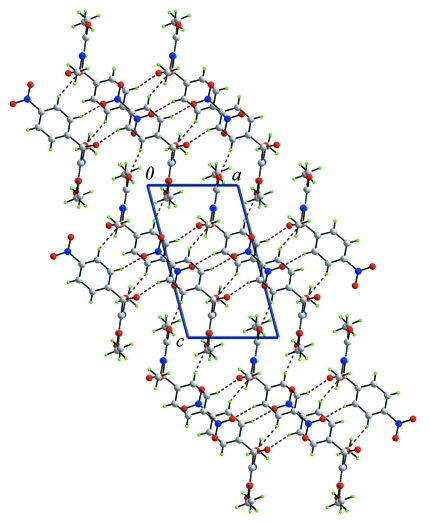
A view along the b axis of the crystal packing of the title compound, highlighting the interdigitation of the ester groups [the C–H···O contacts are shown as brown dashed lines; colour code: O, red; N, blue; C, grey; H, green].

## supplementary crystallographic information

### Comment

In connection with studies involving the synthesis of chiral hydroxyaminoacids and hydroxyaminoalcohols, whereby α-ketomethoxyimino compounds are reduced by sodium borohydride (Corrêa & Moran, 1999) and enantioselectively bio-reduced by whole cells of yeast (Kreutz *et al.*, 1997; Kreutz *et al.*, 2000), the title compound was prepared as an intermediary.

The molecular structure of the title compound is illustrated in Fig. 1, and the geometrical parameters are given in the Supplementary information and the archived CIF. The conformation about the oxime bond [N2═C8 = 1.2790 (14) Å] is *E*. The methoxyimino moiety is effectively co-planar with the ester group, as seen in the sequence of O4-N2-C8-C9, N2-C8-C9-O5 and C8-C9-O6-C10 torsion angles of 177.54 (8), 6.76 (16) and 178.40 (9) °, respectively. While atom C7 lies in the mean plane of the ester group [O4-N2-C8-C7 = 2.17 (14) °], the carbonyl and nitrophenyl groups occupy positions almost orthogonal to the remaining atoms, as seen in the values of the N2-C8-C7-O3 and N2-C8-C7-C4 torsion angles of 99.44 (13) and -77.85 (13) °, respectively. The nitro group and the benzene ring to which it is attached are slightly twisted with respect to one another, with a dihedral angle of 8.54 (8)°. Globally, when viewed down the C7–C8 bond, the carbonyl-O3 atom lies to one side of the central plane and the nitrophenyl group to the other.

The crystal packing is dominated by C–H(aromatic)···O interactions involving the nitro-O2, carbonyl-O3 and oxime-O4 atoms to form layers in the *ab* plane (Table 1 and Fig. 2). These layers stack along [001] with interdigitation of the ethyl ester groups (Fig. 3), and C–H···O interactions involving the ester carbonyl-O5 atom.

The basic C(═O)C(═NOH)C(═O)OC framework in the title compound is comparatively rare with only three other structures reported, namely the recently described *Z*-isomer of the title compound (Caracelli *et al.*, 2010), 2-(hydroxyimino)-3-oxo-3-phenylpropionate, where *E*-conformations are found for each of the independent molecules (Ramos Silva *et al.*, 2004), and benzyl 2-(hydroxyimino)acetoacetate, for which a *Z* conformation is found (Forsyth *et al.*, 2006).

### Experimental

The title compound was prepared following a modified literature method (Buehler, 1967). Silver oxide (1.3 mmol) was slowly added with stirring to a solution of a mixture of ethyl (*E*)- and (*Z*)-2-hydroximino-3-(4-nitrophenyl)-3-oxopropanoate (2.25 mmol) and methyl iodide (5.6 mmol) in CH_2_Cl_2_ (30 ml), and cooled in a ice-water bath. The temperature was then raised to 301 K and the stirring maintained for 1 h. The precipitate formed was filtered off and washed with CH_2_Cl_2_. The solvent was evaporated to afford yellow crystals of a mixture of *E*:*Z* (90:10) isomers in 86% yield. They were separated by TLC chromatography on silica gel with 5% ethyl acetate/hexane. The principal fraction was shown by crystallographic analysis to be the *E* isomer; m.p.  367.6–368.0 K.

### Refinement

The H atoms were placed geometrically (C–H = 0.93–0.97 Å) and refined in the riding model approximation with U_iso_(H) = 1.2U_eq_(C-aromatic) and 1.5U_eq_(C-methyl).

### Crystal data

**Table:**

C_12_H_12_N_2_O_6_	*Z* = 2
*M**_r_* = 280.24	*F*(000) = 292
Triclinic, *P*1	*D*_x_ = 1.478 Mg m^−^^3^
Hall symbol: -P 1	Mo *K*α radiation, λ = 0.71073 Å
*a* = 7.5197 (1) Å	Cell parameters from 5430 reflections
*b* = 7.5793 (1) Å	θ = 26.6–2.9°
*c* = 12.3338 (2) Å	µ = 0.12 mm^−^^1^
α = 83.264 (1)°	*T* = 100 K
β = 73.731 (1)°	Block, pale-yellow
γ = 68.939 (1)°	0.35 × 0.25 × 0.08 mm
*V* = 629.62 (2) Å^3^	

### Data collection

**Table:**

Bruker APEXII CCD diffractometer	2614 independent reflections
Radiation source: fine-focus sealed tube	2310 reflections with *I* > 2σ(*I*)
graphite	*R*_int_ = 0.018
φ and ω scans	θ_max_ = 26.6°, θ_min_ = 1.7°
Absorption correction: multi scan (*SADABS*; Sheldrick, 1996)	*h* = −9→9
*T*_min_ = 0.933, *T*_max_ = 1.000	*k* = −9→9
9325 measured reflections	*l* = −15→15

### Refinement

**Table:**

Refinement on *F*^2^	Primary atom site location: structure-invariant direct methods
Least-squares matrix: full	Secondary atom site location: difference Fourier map
*R*[*F*^2^ > 2σ(*F*^2^)] = 0.031	Hydrogen site location: inferred from neighbouring sites
*wR*(*F*^2^) = 0.088	H-atom parameters constrained
*S* = 1.05	*w* = 1/[σ^2^(*F*_o_^2^) + (0.0456*P*)^2^ + 0.1834*P*] where *P* = (*F*_o_^2^ + 2*F*_c_^2^)/3
2614 reflections	(Δ/σ)_max_ = 0.001
183 parameters	Δρ_max_ = 0.28 e Å^−^^3^
0 restraints	Δρ_min_ = −0.24 e Å^−^^3^

### Special details

**Table:**

Geometry. All s.u.'s (except the s.u. in the dihedral angle between two l.s. planes) are estimated using the full covariance matrix. The cell s.u.'s are taken into account individually in the estimation of s.u.'s in distances, angles and torsion angles; correlations between s.u.'s in cell parameters are only used when they are defined by crystal symmetry. An approximate (isotropic) treatment of cell s.u.'s is used for estimating s.u.'s involving l.s. planes.
Refinement. Refinement of *F*^2^ against ALL reflections. The weighted *R*-factor *wR* and goodness of fit *S* are based on *F*^2^, conventional *R*-factors *R* are based on *F*, with *F* set to zero for negative *F*^2^. The threshold expression of *F*^2^ > 2σ(*F*^2^) is used only for calculating *R*-factors(gt) *etc*. and is not relevant to the choice of reflections for refinement. *R*-factors based on *F*^2^ are statistically about twice as large as those based on *F*, and *R*- factors based on ALL data will be even larger.

### Fractional atomic coordinates and isotropic or equivalent isotropic displacement parameters (Å^2^)

**Table:**

	*x*	*y*	*z*	*U*_iso_*/*U*_eq_	
C1	0.95878 (16)	0.26376 (14)	0.48218 (9)	0.0180 (2)	
C2	0.77279 (16)	0.24949 (15)	0.51100 (9)	0.0190 (2)	
H2	0.7241	0.2000	0.5810	0.023*	
C3	0.66149 (16)	0.31060 (15)	0.43316 (9)	0.0185 (2)	
H3	0.5370	0.3003	0.4498	0.022*	
C4	0.73587 (16)	0.38808 (14)	0.32919 (9)	0.0174 (2)	
C5	0.92289 (16)	0.40168 (15)	0.30321 (9)	0.0195 (2)	
H5	0.9711	0.4539	0.2341	0.023*	
C6	1.03760 (16)	0.33776 (15)	0.37975 (9)	0.0195 (2)	
H6	1.1636	0.3444	0.3628	0.023*	
C7	0.60888 (16)	0.45924 (14)	0.24893 (9)	0.0173 (2)	
C8	0.67583 (15)	0.57310 (15)	0.14594 (9)	0.0175 (2)	
C9	0.75507 (16)	0.49578 (15)	0.02915 (9)	0.0192 (2)	
C10	0.81887 (17)	0.23916 (16)	−0.08355 (9)	0.0209 (2)	
H10B	0.9588	0.2169	−0.1133	0.025*	
H10A	0.7494	0.3237	−0.1353	0.025*	
C11	0.78050 (19)	0.05568 (16)	−0.07074 (10)	0.0251 (3)	
H11B	0.8255	−0.0029	−0.1430	0.038*	
H11C	0.6416	0.0794	−0.0416	0.038*	
H11A	0.8499	−0.0268	−0.0194	0.038*	
C12	0.55476 (19)	1.00856 (16)	0.25880 (10)	0.0243 (3)	
H12C	0.4759	1.0782	0.2084	0.036*	
H12A	0.6827	1.0209	0.2336	0.036*	
H12B	0.4920	1.0575	0.3336	0.036*	
N2	0.65628 (13)	0.74693 (13)	0.15091 (8)	0.0189 (2)	
N1	1.07936 (14)	0.19686 (13)	0.56457 (8)	0.0204 (2)	
O6	0.74864 (12)	0.32311 (11)	0.02858 (6)	0.02006 (19)	
O5	0.81359 (14)	0.58238 (12)	−0.05260 (7)	0.0283 (2)	
O3	0.45219 (12)	0.43609 (11)	0.26469 (7)	0.02198 (19)	
O4	0.57531 (12)	0.81146 (11)	0.26006 (6)	0.0221 (2)	
O1	1.00183 (13)	0.15085 (13)	0.65930 (7)	0.0300 (2)	
O2	1.25168 (13)	0.18865 (13)	0.53367 (7)	0.0285 (2)	

### Atomic displacement parameters (Å^2^)

**Table:**

	*U*^11^	*U*^22^	*U*^33^	*U*^12^	*U*^13^	*U*^23^
C1	0.0223 (6)	0.0154 (5)	0.0157 (5)	−0.0056 (4)	−0.0047 (4)	−0.0012 (4)
C2	0.0233 (6)	0.0175 (5)	0.0144 (5)	−0.0084 (4)	−0.0006 (4)	0.0004 (4)
C3	0.0184 (5)	0.0185 (5)	0.0182 (5)	−0.0086 (4)	−0.0006 (4)	−0.0011 (4)
C4	0.0208 (5)	0.0153 (5)	0.0154 (5)	−0.0070 (4)	−0.0021 (4)	−0.0012 (4)
C5	0.0215 (5)	0.0202 (5)	0.0156 (5)	−0.0092 (4)	−0.0014 (4)	0.0021 (4)
C6	0.0180 (5)	0.0209 (5)	0.0194 (5)	−0.0087 (4)	−0.0020 (4)	0.0001 (4)
C7	0.0196 (5)	0.0155 (5)	0.0154 (5)	−0.0069 (4)	−0.0005 (4)	−0.0020 (4)
C8	0.0168 (5)	0.0200 (5)	0.0168 (5)	−0.0082 (4)	−0.0042 (4)	0.0016 (4)
C9	0.0192 (5)	0.0209 (5)	0.0182 (5)	−0.0079 (4)	−0.0048 (4)	0.0009 (4)
C10	0.0250 (6)	0.0227 (5)	0.0151 (5)	−0.0090 (4)	−0.0040 (4)	−0.0010 (4)
C11	0.0329 (7)	0.0234 (6)	0.0217 (6)	−0.0122 (5)	−0.0080 (5)	−0.0004 (5)
C12	0.0306 (6)	0.0174 (5)	0.0252 (6)	−0.0089 (5)	−0.0062 (5)	−0.0017 (4)
N2	0.0200 (5)	0.0212 (5)	0.0156 (5)	−0.0081 (4)	−0.0036 (4)	−0.0003 (4)
N1	0.0239 (5)	0.0198 (5)	0.0178 (5)	−0.0080 (4)	−0.0050 (4)	−0.0001 (4)
O6	0.0259 (4)	0.0197 (4)	0.0152 (4)	−0.0102 (3)	−0.0029 (3)	−0.0004 (3)
O5	0.0416 (5)	0.0267 (4)	0.0174 (4)	−0.0187 (4)	−0.0005 (4)	0.0013 (3)
O3	0.0217 (4)	0.0246 (4)	0.0219 (4)	−0.0121 (3)	−0.0051 (3)	0.0028 (3)
O4	0.0302 (4)	0.0188 (4)	0.0166 (4)	−0.0104 (3)	−0.0016 (3)	−0.0017 (3)
O1	0.0309 (5)	0.0414 (5)	0.0160 (4)	−0.0132 (4)	−0.0049 (4)	0.0057 (4)
O2	0.0245 (4)	0.0382 (5)	0.0269 (5)	−0.0153 (4)	−0.0094 (4)	0.0058 (4)

### Geometric parameters (Å, °)

**Table:**

C1—C6	1.3839 (15)	C9—O5	1.2020 (14)
C1—C2	1.3839 (16)	C9—O6	1.3278 (13)
C1—N1	1.4753 (14)	C10—O6	1.4670 (13)
C2—C3	1.3798 (16)	C10—C11	1.5011 (16)
C2—H2	0.9300	C10—H10B	0.9700
C3—C4	1.4000 (15)	C10—H10A	0.9700
C3—H3	0.9300	C11—H11B	0.9600
C4—C5	1.3910 (16)	C11—H11C	0.9600
C4—C7	1.4889 (15)	C11—H11A	0.9600
C5—C6	1.3851 (16)	C12—O4	1.4442 (13)
C5—H5	0.9300	C12—H12C	0.9600
C6—H6	0.9300	C12—H12A	0.9600
C7—O3	1.2121 (14)	C12—H12B	0.9600
C7—C8	1.5194 (14)	N2—O4	1.3797 (12)
C8—N2	1.2790 (14)	N1—O1	1.2229 (12)
C8—C9	1.4965 (15)	N1—O2	1.2244 (13)
			
C6—C1—C2	123.25 (10)	O6—C9—C8	111.19 (9)
C6—C1—N1	118.25 (10)	O6—C10—C11	107.57 (9)
C2—C1—N1	118.51 (9)	O6—C10—H10B	110.2
C3—C2—C1	118.23 (10)	C11—C10—H10B	110.2
C3—C2—H2	120.9	O6—C10—H10A	110.2
C1—C2—H2	120.9	C11—C10—H10A	110.2
C2—C3—C4	120.00 (10)	H10B—C10—H10A	108.5
C2—C3—H3	120.0	C10—C11—H11B	109.5
C4—C3—H3	120.0	C10—C11—H11C	109.5
C5—C4—C3	120.30 (10)	H11B—C11—H11C	109.5
C5—C4—C7	121.09 (10)	C10—C11—H11A	109.5
C3—C4—C7	118.59 (10)	H11B—C11—H11A	109.5
C6—C5—C4	120.29 (10)	H11C—C11—H11A	109.5
C6—C5—H5	119.9	O4—C12—H12C	109.5
C4—C5—H5	119.9	O4—C12—H12A	109.5
C1—C6—C5	117.91 (10)	H12C—C12—H12A	109.5
C1—C6—H6	121.0	O4—C12—H12B	109.5
C5—C6—H6	121.0	H12C—C12—H12B	109.5
O3—C7—C4	122.79 (10)	H12A—C12—H12B	109.5
O3—C7—C8	119.12 (10)	C8—N2—O4	111.55 (9)
C4—C7—C8	118.03 (9)	O1—N1—O2	123.89 (10)
N2—C8—C9	114.45 (9)	O1—N1—C1	118.03 (9)
N2—C8—C7	122.66 (9)	O2—N1—C1	118.08 (9)
C9—C8—C7	122.72 (9)	C9—O6—C10	114.57 (8)
O5—C9—O6	125.40 (10)	N2—O4—C12	108.40 (8)
O5—C9—C8	123.40 (10)		
			
C6—C1—C2—C3	0.61 (16)	O3—C7—C8—C9	−75.55 (14)
N1—C1—C2—C3	−179.38 (9)	C4—C7—C8—C9	107.17 (12)
C1—C2—C3—C4	−1.27 (15)	N2—C8—C9—O5	6.76 (16)
C2—C3—C4—C5	0.82 (16)	C7—C8—C9—O5	−177.87 (11)
C2—C3—C4—C7	−177.73 (9)	N2—C8—C9—O6	−172.26 (9)
C3—C4—C5—C6	0.35 (16)	C7—C8—C9—O6	3.10 (14)
C7—C4—C5—C6	178.85 (10)	C9—C8—N2—O4	177.54 (8)
C2—C1—C6—C5	0.52 (16)	C7—C8—N2—O4	2.17 (14)
N1—C1—C6—C5	−179.49 (9)	C6—C1—N1—O1	172.19 (10)
C4—C5—C6—C1	−0.99 (16)	C2—C1—N1—O1	−7.82 (14)
C5—C4—C7—O3	173.89 (10)	C6—C1—N1—O2	−8.63 (15)
C3—C4—C7—O3	−7.58 (16)	C2—C1—N1—O2	171.36 (10)
C5—C4—C7—C8	−8.93 (15)	O5—C9—O6—C10	−0.60 (16)
C3—C4—C7—C8	169.60 (9)	C8—C9—O6—C10	178.40 (9)
O3—C7—C8—N2	99.44 (13)	C11—C10—O6—C9	−173.74 (9)
C4—C7—C8—N2	−77.85 (13)	C8—N2—O4—C12	−179.06 (9)

### Hydrogen-bond geometry (Å, °)

**Table:**

*D*—H···*A*	*D*—H	H···*A*	*D*···*A*	*D*—H···*A*
C2—H2···O4^i^	0.93	2.56	3.3853 (14)	148
C3—H3···O2^ii^	0.93	2.50	3.3950 (16)	162
C5—H5···O5^iii^	0.93	2.35	3.1856 (14)	150
C6—H6···O3^iv^	0.93	2.46	3.3514 (16)	160

Symmetry codes: (i) −*x*+1, −*y*+1, −*z*+1; (ii) *x*−1, *y*, *z*; (iii) −*x*+2, −*y*+1, −*z*; (iv) *x*+1, *y*, *z*.
